# Sterilization incentives and associated regret among ever married women in India, NFHS, 2015–16

**DOI:** 10.1186/s12913-022-08401-8

**Published:** 2022-08-19

**Authors:** Anjali Bansal, Laxmi Kant Dwivedi, P. Shirisha

**Affiliations:** 1 International Institute for Population Sciences, Govandi St Rd, Deonar, Mumbai, 400088 India; 2 Department of Survey Research & Data Analytics, Govandi St Rd, Deonar, Mumbai, 400088 India; 3 Department of Humanities and Social Sciences, Humanities and Science Block, IIT Madras, Chennai, Tamil Nadu India

**Keywords:** Sterilization Regret, Incentives, DHS, India

## Abstract

**Objective:**

Sterilization is the only family planning method that involves relatively large amount compensation. So, the study attempts to examine the role of incentives received against the sterilization procedures on the reporting of sterilization regret in India.

**Methods:**

The study used data from the fourth round of National Family Health Survey, 2015–16, which gathered the information on sterilization regret from 1,94,207 ever-married women. Multivariate logistic analysis and predicted probabilities approach was used to study the effect of compensation received on the sterilization regret in India.

**Results:**

Results show that women who have received compensation were 33% less likely to report sterilization regret. It was found that 70% of women who undergone sterilization in public facility didn’t incur any expenditure, rather received incentives. It is observed that women who had undergone operation in private facility spent a large amount than women who had done their operation in public facility. The regret in the private facility mainly results from high out of pocket expenditure on sterilization procedures. Around eight percent of women regretted getting sterilized in a private hospital and received some compensation amount, vis a vis the six percent who regretted undergoing sterilization in public facility and received compensation.

**Conclusion:**

The study calls for a need to standardize the cost of sterilization procedure in India's health facilities. A good alternative for reducing the cost could be Public–Private Partnership.

## Introduction

In India during the 1950s, policymakers perceived that the high population growth rate could be detrimental to country’s economic growth and development. As a result, first Five-year Development Plan (1952–57) recognized the urgency to deal with the problem of family planning and population control. Despite the concerted efforts, the birth rate remained more or less the same, at 41 per 1000 in 1951 till 1971. Thereafter, the government adopted the policy of persuasion through coerced sterilization in the fifth Five-year plan (1974–79) [1]. This caused massive political upheaval, as a result a new government was formed which shifted the family planning programme to family welfare approach.

In the recent years, there has been an alarming decline in male sterilization, to the extent of which, now the procedure being practically non-existent (74% in 1970 to 1.2% in 2015), [2, 3] while female sterilization emerged as the only permanent method practised in the country among the married women. According to the latest estimates by the United Nations (UN), 24% of married or in-union women in the world relied only on female sterilization [4]. In India, according to NFHS-5, among the currently married women 50% are using modern contraceptive methods, while 38% were depending only on female sterilization [5]. As sterilization is an irreversible process, women should be informed about its consequences and it should be performed only after the women has achieved desired parity else it may lead to sterilization regret. Though sterilization has improved the lives of many, but a sizeable proportion of women still regret the undergoing procedure owing to different reasons.

In India, sterilization regret has increased to two percent point in a decade, from five per cent in 2005–06 to seven per cent in 2015–16, which roughly translates to around 280,000 women per year [6]. Earlier have tried to capture the reasons for sterilization regret, and found that the quality of services and type of health provider have a direct bearing on the sterilization regret along with several socio-economic and demographic variables [6–8]. Mostly women who got sterilized at a younger age (less than 30) were about twice as likely to report regret than older women [9, 10]. Quality issues in the government facilities during the sterilization procedure has been cited as the main reason for the sterilization regret in India [11, 12] Many studies also suggest that use of a single method predominantly, indicates insufficient choices [13, 14] which often leads to regret.

The phenomena of sterilization regret has been studied extensively in developed countries as compared to the developing countries [15–21]. In India a few studies used the large scale data to explore the sterilization regret pertaining to different socio-demographic factors and also quality of care issues during the procedure [11, 22], but none of the studies have explored the association of sterilization regret and cost of compensation received against the procedure. Sterilization is the only method in India which involves huge compensation cost and the cost of compensation differes for each state based on the their fertility rates. The compensation cost in India varies from 3.5 to 15.5 USD based on the fertility rates. In the high focus state, the compensation amount is about 15.5 USD per vasectomy and 8.5 USD per tubectomy, whereas, in the non-high focus state, the compensation received from tubectomy is 3.5 USD (for non-Below Poverty line, Scheduled caste and tribe), the vasectomy compensation was the same as in the high focus states [23]. So, in this study we hypothesized that since sterilization is the only family planning method which involves compensation, so many times couples may undergo sterilization to recieve the compensation and later regret it. Specifically in this study our endeavour is to identify the association between cost involved in the procedure (sterilization operation expenditure, compensation received, and out of pocket expenditure) and the sterilization regret among ever married women in India. An attempt has also been made to establish the linkages of compensation received with reporting of sterilization regret by public and private facility.

## Methods

### Data 

The data used for the analysis is from the fourth round of the NFHS-4 (2015–2016). NFHS is a nationally representative cross-sectional survey which includes representative samples of the household throughout India. The survey provides national, state and district level estimates of demographic and health parameters as well as data on various socio-economic and program dimensions. Stratified, two-stage sampling method is mostly used in all DHS surveys to obtain a representative sample of households. Probability proportional to size (PPS) sampling method was used to select the villages in rural and Census Enumeration blocks (CEBs) in urban areas from all states and Union Territories (the detailed sampling method is available here [3]). The data on the year of sterilization, cost of female sterilization, and amount of compensation received for all the ever-married sterilized women were included for the first time in NFHS-4.

### Sample

The fourth round collected data from 699,686 ever-married women aged 15–49 in 601,509 households. Since the objective was to assess the sterilization regret due to the compensation received in the sterilization operations, we have restricted our sample to those ever-married women who were sterilized at the time of the survey. In NFHS-4 194,713 ever-married women reported of being sterilized at the time of the survey. In the Indian setting, it is usually not possible for a woman to undergo sterilization before having a child, so we have excluded 505 women from the analysis who have reported zero parity at the time of the survey. The effective sample size in NFHS-4 consists of 1, 94,207 ever-married women.

We have also computed the out of Pocket (OOP) expenditure incurred in the sterilization operations using the cost spent in the sterilization operations and amount received against compensation. The compensation received and amount paid for female sterilization was truncated at 99.5 percentile [24]. The OOP is defined as the total amount spent on sterilization with respect to amount received as compensation. The data related to a data error, don't know (DK), and missing was adjusted before computing the OOP [25]. Out of the total 1,94,207 ever-married women, 8,651 women reported don't know about the sterilization cost, and 3,664 did not know about the amount of compensation received, so after adjusting, an effective sample size of 181,892 ever-married women was used to compute the OOP for the sterilization operation in India.

### Outcome variable

Ever married women who have undergone sterilization were asked about whether they regretted of being sterilized. A dichotomous variable was constructed to assess the sterilization regret, where 1 "included women who regretted about the procedure" and 0 otherwise.

### Independent variables

A growing literature indicates that various socio-economic, demographic and other factors influence the sterilization regret among women in both developed and developing countries. So based on literatures, we have selected out independent variables, type of facility, compensation received, quality of care during and post sterilization, information on sterilization, and socio-economic and demographic characteristics.

Detailed description of the variables were as follows. Age at sterilization (less than 25, 25–29, and 30 or more), parity at sterilization (1, 2–3, and 3 or more), year since sterilization (less than 2, 2–5, 6–9, 10 or more), type of health facility (public, private and others), and no one said sterilization would mean no more children (yes, and no), quality of care during and immediately after sterilization (very good, all right, not so good and bad), the cost variable included as compensation received (yes, no), and OOP (No cost incurred, less than 3000, more than 3000). Child related factors were, sex composition of living children (only son, only daughter, both son and daughter), child loss (no loss, loss before sterilization, loss after sterilization), overall loss (no loss, one loss, two-loss, more than two-loss). The other variables included, loss before geographic region (North, Central, East, North East, West, South), place of residence (rural, urban). The household variables included are caste (Scheduled Caste (SC), Scheduled Tribe (ST), Other Backward Classes (OBC), others), religion (Hindu, Muslims, Christian, others), wealth quintile (poorest, poorer, middle, richer, richest). The individual-level variables included are the age of women (15–19, 20-24, 25–29, 30–34, 35–39, 40–44, 45-49), women's education (no education, primary, secondary, higher).

### Statistical analysis

Descriptive analysis, univariate and multivariable logistic regression analysis were used to determine the factors associated with reporting of sterilization regret. Based on the growing literature on sterilization, we have selected 18 covariates, then we performed univariate logistic analysis to select independent variables for the multivariable model. Factors found significant in the univariate analysis (*P*-value < 0.05) (not shown) were included in the multivariable model.

For the multivariable analysis, three models were constructed—Model 1, regression analysis was run for ever-married women which assessed the associations of sterilization regret controlled for all the independent variables which were found significant in univariate logistic regression analysis except for the variable “whether compensation received for sterilization”. Model 2 – was adjusted model assessing the sterilization regret controlling for all significant independent variables, including compensation received and Model 3 assessed the sterilization regret controlling for all significant independent variables, including the out of pocket expenditure.

To determine the effect of compensation and type of health facility on the sterilization regret, two interaction terms were created, by interacting one category of compensation received with two categories of health facility, and multivariable logistic regression was performed. Also, to find the association of cost with the type of service provider, four interaction terms were created, by interacting two categories of OOP with two categories of type of health facility. The model in the study is defined as:

$$log(Y)=a+b_1X_i+b_2X_2+b_3X_iX_2$$

Where log(Y) is log of odds of sterilization regret among ever married women, a is the intercept and b_1_ and b_2_ are the coefficients associated with each independent variable, b_3_ is the coefficient associated with the interaction term between X_1_ and X_2_.

The predicted probabilities were also calculated based on the logistic regression model relating to interactions between compensation received, and, type of health facility and in the model-3, OOP and type of health facility. The standard errors for all the logistic regression was adjusted for the clusters. The details and methodology of the adjustment of cluster is available elsewhere [26].

All analyses were completed using Stata version 15.0, and results were reported at a 5% level of significance.

## Results

### Socio-economic and demographic trends of sterilization regret in India

Table 1 represents the possible pathways of sterilization regret among ever-married women in India by different covariates. Cost was an important factor for sterilization regret, around 8% of women who didn’t receive any compensation or incurred an OOP expenditure of more than Rs.3000 regretted of being sterilized. Also, 15% of the women who lost their child post-sterilization regretted the more uneducated urban women from poor wealth quintiles, residing in southern regions regretted the sterilization procedures more than their counterparts.

**Table 1:** Trends of sterilization regret among ever-married women in India by type of facility, compensation received, quality of care during and post sterilization, information given on sterilization, and socio-economic and demographic characteristics, 2015–16

Variables	NFHS-4 (2015–16)
**Age at sterilization**	
< 25	7.01
25–29	6.54
> = 30	6.51
**Year since sterilization**	
< 2	6.48
2–5	6.97
6–9	7.20
More than 10 years	6.83
**Parity at sterilization**	
1	10.74
2–3	6.86
> 3	5.79
**Told sterilization would mean no more children**	
No	5.55
Yes	6.99
**Compensation received**	
No	7.50
Yes	6.32
**Out of Pocket Expenditure Incurred**	
No OOP	6.75
Less than 3000	7.26
More than equal to 3000	7.67
**Quality of care**	
Very good	7.61
All right	5.27
Not so good	10.40
Bad	18.25
**Type of health facility**	
Public	6.69
Private	6.77
Others	2.07
**Sex Composition**	
Only Son	7.59
Only Daughter	10.51
Both	5.91
**Child loss**	
No loss	6.81
Before Sterilization	7.36
After Sterilization	14.61
**Child loss overall**	
No loss	6.66
1 child loss	7.32
2 child loss	5.32
More than 2 loss	5.91
**Currently married**	
Yes	6.72
No	6.70
**Age of women**	
15–19	8.70
20–24	7.24
25–29	6.49
30–34	6.76
35–39	6.52
40–44	6.58
45–49	6.19
**Educational Status**	
No education	6.34
Primary	6.01
Secondary	7.14
Higher	6.93
**Caste**	
SC	6.61
ST	6.67
OBC	7.04
Others	6.06
**Religion**	
Hindu	6.55
Muslim	8.76
Christin	7.61
Others	3.98
**Wealth Quintile**	
Poorest	6.06
Poorer	6.61
Middle	6.69
Richer	7.25
Richest	7.04
**Region**	
North	5.29
Central	6.36
East	6.76
North East	5.47
West	5.44
South	8.15
**Place of residence**	
Urban	6.92
Rural	6.61
** Total**	**6.90**

### Amount paid, compensation received and OOP for female sterilization in India

**Fig. 1:**
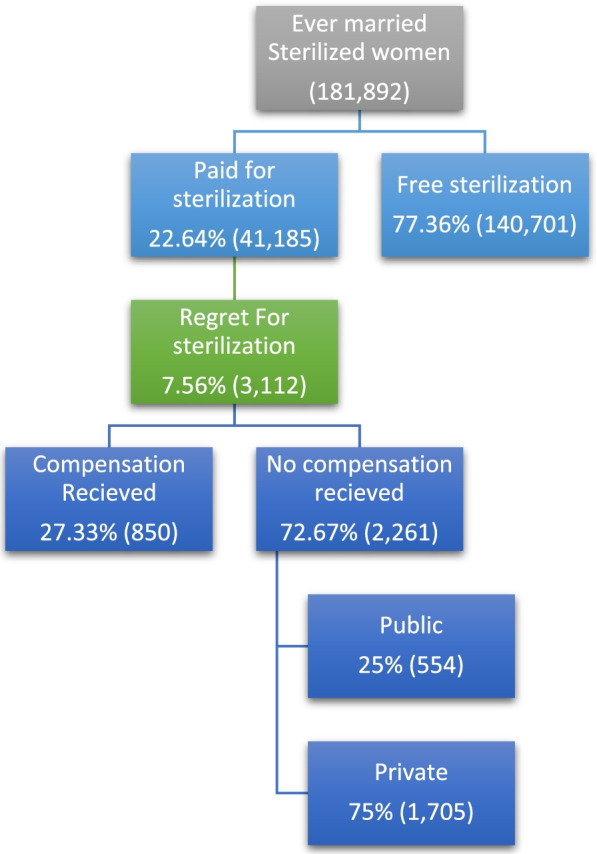
Systematic representation of percentage ever married women who have undergone for sterilization by cost, compensation received and type of health facilities

To provide an insight on how the cost affected the sterilization regret in India, we have presented the systematic representation of ever married women who reported the amount paid or received for sterilization operation by sterilization regret and health facility (Fig. 1). Out of the 181,892 ever married, 23% paid for their sterilization, and 77% had their sterilization free of cost. Of those who paid, 75% of the women has done her sterilization in private facility.

From Table 2, it is observed that mean sterilization expenditure incurred by women in India was INR 1244 (15.94 USD), (95% CI (1221.0 – 1267.6)) while the distribution of expenditure was completely different by type of health facility. The expenditure was only INR 270 (3.46 USD) ( 95% CI (259.9 – 280.1) when sterilization was performed in a public facility, while it was INR 8241 (105.57 USD) (95% CI (8100.9–8381.6)) when performed in private facility, which is almost 30 times more than public facility.

**Table 2:** Mean Sterilization expenditure, compensation received and Out of Pocket expenditure for sterilization opeartion by type of health facility for ever married sterilized women in India, NFHS, 2015–16

	**N**	**Mean Sterilization Expenditure**	**Mean Compensation Received**	**Out of Expenditure**
Public	1,53,423	270.0 (259.9,280.1)	382.7 ( 380.3,385.1)	-112.7 (-123.1, -102.3)
Private	28,152	8240.8 (8100.0,8381.6)	50.4 (45.6,55.2)	8190.4 (8049.5,8331.4)
Others	318	1444.9 (801.2,2088.6)	278.4 (226.5,330.4)	1166.5 (513.6,1819.4)
**Total**	**1,81,892**	**1244.3 (1221.0,1267.6)**	**342.0 (339.8,344.2)**	**902.3 (878.6,926.0)**

### Multivariate Logistic analysis

In multivariable logistic regression analysis, several aspects of sterilization regret were observed using three different models (Table 3).

**Table 3:** Adjusted odds ratios (with 95% CI) from binary logistic regressions examining the sterilization regret among ever married women by selected covariates for ever married women in India, NFHS-4

	**NFHS-4 (2015–16)**		
**Variables**	**Model I**	**Model II**	**Model III**
	**AOR (95% CI)**	**AOR (95% CI)**	**AOR (95% CI)**
**Age at sterilization**			
< 25	1.00	1.00	1.00
25–29	0.96 (0.90 1.02)	0.96 (0.90 1.02)	0.95 (0.89 1.02)
> = 30	0.96 (0.89 1.04)	0.96 (0.88 1.04)	0.97 (0.89 1.06)
**Year since sterilization**			
Less than 2	1.00	1.00	1.00
2–5	1.11 (1.00 1.23)	1.11*(0.99 1.23)	1.12*(1.01 1.25)
6–9	1.17*(1.05 1.30)	1.17*(1.05 1.30)	1.20*(1.07 1.34)
10 +	1.09* (1.02 1.20)	1.07 (0.97 1.19)	1.12*(1.01 1.24)
**Parity at sterilization**			
1	1.00	1.00	1.00
2–3	0.72*(0.62 0.83)	0.72*(0.62 0.83)	0.71*(0.61 0.84)
> 3	0.61*(0.52 0.72)	0.62*(0.52 0.73)	0.61*(0.51 0.72)
**Told sterilization would mean no more children**			
No	1.00	1.00	1.00
Yes	1.41*(1.31 1.52)	1.42*(1.32 1.53)	1.38*(1.27 1.51)
**Compensation received**			
No	-	1.00	-
Yes	-	0.87*(0.81 0.93)	-
**Quality of care**			
Very good	1.00	1.00	1.00
All right	0.73*(0.69 0.77)	0.73*(0.69 0.77)	0.72*(0.68 0.78)
Not so good	1.28*(1.13 1.45)	1.27*(1.12 1.44)	1.26*(1.09 1.45)
Bad	3.08*(2.26 4.19)	3.03*(2.23 4.12)	2.80*(2.08 3.78)
**Type of health facility**			
Public	1.00	1.00	1.00
Private	0.92 (0.85 1.02)	0.85*(0.77 0.93)	0.76*(0.66 0.88)
Others	0.56 (0.3 1.06)	0.53*(0.28 1.01)	0.47*(0.23 0.99)
**Sex Composition**			
Only Son	1.00	1.00	1.00
Only Daughter	1.22*(1.10 1.35)	1.22*(1.1 1.35)	1.22*(1.1 1.36)
Both	0.76*(0.71 0.81)	0.76*(0.71 0.81)	0.78*(0.73 0.84)
**Child loss**			
No loss	1.00	1.00	1.00
Before Sterilization	1.23*(1.13 1.34)	1.21*(1.12 1.31)	2.21*(1.2 4.1)
After Sterilization	2.17*(1.16 4.06)	2.34*(1.22 4.49)	1.20*(1.1 1.3)
**Currently married**			
Yes	1.00	1.00	1.00
No	0.86*(0.76 0.98)	0.88*(0.78 1.00)	1.18*(1.04 1.35)
**Educational Status**			
No education	1.00	1.00	1.00
Primary	1.03 (0.95 1.11)	1.03 (0.95 1.12)	1.04 (0.96 1.13)
Secondary	1.09 (1.01 1.17)	1.09*(1.01 1.17)	1.09*(1.01 1.17)
Higher	1.02 (0.87 1.2)	1.02 (0.87 1.20)	1.06 (0.88 1.27)
**Caste**			
SC	1.00	1.00	1.00
ST	1.05 (0.95 1.17)	1.05 (0.95 1.16)	1.05 (0.93 1.19)
OBC	0.96 (0.9 1.04)	0.96 (0.90 1.03)	0.96 (0.89 1.05)
Others	1.00 (0.91 1.09)	0.99 (0.91 1.09)	1.00 (0.90 1.11)
**Religion**			
Hindu	1.00	1.00	1.00
Muslim	1.38*(1.24 1.52)	1.37*(1.23 1.52)	1.36*(1.20 1.55)
Christin	0.94 (0.78 1.12)	0.93 (0.78 1.12)	0.87 (0.71 1.07)
Others	0.89 (0.74 1.08)	0.89 (0.73 1.07)	0.85 (0.69 1.03)
**Wealth Quintile**			
Poorest	1.00	1.00	1.00
Poorer	1.05 (0.97 1.14)	1.05 (0.96 1.14)	1.06 (0.97 1.16)
Middle	0.99 (0.91 1.09)	0.99 (0.90 1.08)	0.99 (0.9 1.10)
Richer	0.98 (0.89 1.09)	0.98 (0.88 1.08)	0.98 (0.87 1.10)
Richest	0.93 (0.82 1.05)	0.92 (0.81 1.04)	0.93 (0.81 1.07)
**Region**			
North	1.00	1.00	1.00
Central	1.25*(1.15 1.35)	1.27*(1.18 1.38)	1.26*(1.14 1.40)
East	1.21*(1.1 1.33)	1.23*(1.11 1.35)	1.19*(1.06 1.34)
North East	0.89 (0.75 1.05)	0.89 (0.75 1.06)	0.94 (0.75 1.17)
West	0.77*(0.69 0.86)	0.77*(0.69 0.86)	0.78*(0.68 0.89)
South	1.38*(1.28 1.5)	1.39*(1.28 1.51)	1.41*(1.26 1.57)
**Place of residence**			
Urban	1.00	1.00	1.00
Rural	1.04 (0.97 1.11)	1.04 (0.97 1.12)	1.06 (0.96 1.16)
**Out of Pocket expenditure incurred in sterilization operation**	-	-	
No expenditure			
Less than 3000			1.21*(1.07 1.36)
More than equal to 3000			1.31*(1.12 1.53)

^*^p < 0.05

From model 1, we found that women sterilized at higher parity were found to be regretting lesser about their decision (AOR = 0.61, 95% CI (0.52–0.72)) than women who sterilized at lower parity. Women reporting bad quality had three folds higher odds of reporting regret than those reporting very good quality during sterilization. It was found that the type of health facility has no significant role in determining sterilization regret in India. The gender composition of the children was also associated with sterilization regret, where the respondents having only daughters were 1.22 times odds more likely to report regret than those having only sons. Child loss also impacted the sterilization regret, it was found that women who have lost a child after sterilization had 2.17 higher odds than those who did not report any child loss. The individual and household level factors also showed association with sterilization regret, Muslims were more likely to report regret (AOR = 1.38, 95% CI(1.24 = 1.52)) than Hindus. Geographic regions also had a significant impact on sterilization regret, women from the North had 0.77 times lesser odds of reporting regret than those in the west. Women living in Eastern and Central regions were 1.21 and 1.25 times odds more likely to report regret respectively.

Results from model 2 were similar to Model 1, with respect to all the socio-demographic and child related factors. However, in model 2, unlike model 1, the type of facility was found to be a significant factor when the component of compensation received against sterilization operation was included in the model. We found that after controlling for all other variables, women who have received compensation were 0.87 times odds less likely to report sterilization regret compared to those who received incentives. In model 3, after including the categories of OOP against sterilization operation, and adjusting for all the covariates, it was found that women who had incurred OOP more than equal to 3000/- were 31% more likely to report sterilization regret compared to those who did not incur any expenditure ((AOR = 1.31, 95% CI (1.12,153)).

### Results from predicted probabilities

We also estimated the predicted probabilities of sterilization regret for various type of health facility, compensation received and OOP for ever-married women in India as presented in Table 4. The predicted probabilities confirmed the results from the logistic analysis, despite of receiving compensation, eight percent of the women regretted undergoing sterilization in a private facility vis a vis six per cent of those who underwent sterilization in a public health facility. Also, the OOP expenditure was a significant factor associated with sterilization regret in India. The regret increases with the cost spent in the sterilization procedure. Only seven percent of women regretted the sterilization in public facility despite of no OOP for the procedure, in private facility, their proportion was almost eight percent (where OOP was equal to or more than Rs.3000).

**Table 4:** Predicted probability of sterilization regret from logistic regression analysis for different categories of type of health facility, compensation received, and out of pocket expenditure for sterilization among ever married women, India, 2015–16

Predicted Probabilities	Mean
Public facility and not received compensation	0.070 (0.070–0.071)
Public facility and received compensation	0.064 (0.064–0.064)
Private facility and not received compensation	0.066 (0.066–0.066)
Private facility and received compensation	0.077 (0.076–0.079)
Other facility and not received compensation	0.038 (0.036–0.040)
Other facility and received compensation	0.040 (0.037–0.043)
Public facility and no expenditure incurred for sterilization	0.067 (0.067–0.067)
Public facility and incurred less than INR 3000 as OOP	0.089 (0.088–0.090)
Public facility and incurred more than equal to INR 3000 as OOP	0.117 (0.117–0.119)
Private facility and no expenditure incurred for sterilization	0.076 (0.075–0.078)
Private facility and incurred less than INR 3000 as OOP	0.062 (0.061–0.063)
Private facility and incurred more than equal to INR 3000 as OOP	0.077 (0.076–0.078)
Other facility and no expenditure incurred for sterilization	0.036 (0.034–0.039)
Other facility and incurred less than INR 3000 as OOP	0.075 (0.066–0.084)
Other facility and incurred more than equal to INR 3000 as OOP	0.009 (0.007–0.010)

## Discussion

The general picture that emerged from the analysis is that the demand for modern contraceptives remained unchanged since 2005–06 in India, and the female sterilization emerged as the most pre-dominant method among all, and mostly practised in the public sector. Since the 1950s, the demand was satisfied mainly by the public sector, but for the last two decades the contribution of the private sector has increased significantly [27]. In India out of the total expenditure around 85 percent is spent on family planning programmes, where 96% of the money is paid towards compensation in female sterilization, one per cent on spacing methods [28].

Since a huge amount is spent on incentives, therefore we have attempted to examine how does the compensation received affect the sterilization regret among ever-married women in India. Over a decade, the sterilization regret has increased to two percentage points since 2005–06 [3, 29]. Almost all the variables included in the multivariable analysis, have shown a considerable association in the sterilization regret since the last decade. Contrary to previous literature based on sterilization regret, we have found that the type of facility has no role in determining the sterilization regret in India [6, 11], as the regret was same for both public and private sector (seven per cent). Previous literatures on sterilization regret has found that the quality of care in public facility results in the regret among women in India [6, 11, 30, 31] but rather we found that the cost of sterilization operation and compensation received against it, were important determinant of sterilization regret than the quality of health facility. Those women who received compensation against the sterilization operations were less likely to regret the procedure. Also, results from an interacted model found that women who undergo sterilization in a private facility and received compensation were more likely to regret because of the huge cost of sterilization in private facility and less compensation or no compensation received from it. The predicted probability also confirms that women who undergo sterilization in a private facility and received compensation amount reported more sterilization regret than those who underwent sterilization in a public facility and have received compensation amount. Also, it was found that the higher proportion of women reported sterilization regret in public facility when they were spending money from their pockets as a OOP for the procedure. Among the health facility there exists a huge difference in the amount spent on sterilization expenditure. In the private facility the cost incurred for the sterilization operation was 30 times more than public facility, with a meagre compensation of INR 50 (0.64 USD) received against the cost. Similar to our findings, studies from different countries on expenditure incurred on family planning procedures found that in India, only 6% women incurred OOP in public facilities for family planning [32], while majority of those incurring OOP were those who availed the services in a private facility.

Government has always adopted an incentives based strategy to reduce or increase a maternal and child health care indicators. It was found that for reduction of maternal deaths demand side financing like conditional cash transfers have a played an important role [33]. Also, for increasing the acceptance of contraception uptake, well designed service delivery strategies are effective [34–37]. But sometimes for getting these incentives, individuals go through the procedure which they later regret. A study in Rajasthan mentioned that because of the massive incentives, husbands were pushing their wives for the routine process [38] Despite of the ban by Supreme court on sterilization camps, camps were being conducted and massive compensations were being offered to both men and women [39]. Such lucrative offers attracted women to undergo sterilization who eventually regretted the decision. Another study from India revealed that the cost incurred to undergo sterilization operations in the private facilities is huge [24], which results in regret.

Although the study findings offer important insights but it is subjected to certain limitations, the cost estimated in the study does not includes cost for loss of wages, transport facility, and cost of hospital stay, and laboratory fees for test related to operations as NFHS does not provide the data for the same. Also, the study failed to capture all the dimensions of quality of care as suggested by Bruce and Jain framework due to data limitations [40, 41].

## Conclusions

The study concluded that around seven percent of women were regretted about their decision of sterilization according to NFHS-4 (2015–16). Though many socio-economic and demographic factors had influenced the regret, the compensation amount received, and the amount spent in the sterilization operation by the type of service provider significantly influenced the trend of sterilization regret in India. This calls for the need to standardize the cost spent in the sterilization procedure in health facilities especially in the private facility where the cost was found to be huge (105.59 USD). Though government provides some incentives for the cost incurred, but it is meagre when compared to the cost incurred. Although the magnitude of cost of the incentives are typically more efficacious, but government can adopt the public private partnership (PPP) which can help to reduce the huge cost of sterilization in private facilities.

## Data Availability

The datasets analysed during the current study are from National Family Health Survey (NFHS) for India. The data is freely available from the DHS website—The DHS Program—India: Standard DHS, 2015–16 Dataset.
